# Antiretroviral therapy initiated during acute infection in women with HIV-1 clade C reduces anti-Tat antibody production and lowers CD8+ T cell activation

**DOI:** 10.3389/fimmu.2025.1564960

**Published:** 2025-06-18

**Authors:** Thandeka I. Kubheka, Kewreshini Naidoo, Kavidha Reddy, Thumbi Ndung’u, Nompumelelo P. Mkhwanazi

**Affiliations:** ^1^ HIV Pathogenesis Program, The Doris Duke Medical Research Institute, School of Laboratory Medicine and Medical Sciences, College of Health Sciences University of KwaZulu-Natal, Durban, South Africa; ^2^ Africa Health Research Institute, Durban, South Africa; ^3^ Ragon Institute of Massachusetts General Hospital, Massachusetts Institute of Technology and Harvard University, Cambridge, MA, United States; ^4^ Division of Infection and Immunity, University College London, London, United Kingdom

**Keywords:** ART, HIV-1 Tat, Tat antibodies, early treated HIV, ELISA - enzyme-linked immunosorbent assay

## Abstract

**Introduction:**

The HIV-1 Tat protein is essential for virus replication and spread and is therefore a potential target for anti-HIV therapy. Anti-Tat antibodies have been shown to slow HIV disease progression and improve antiretroviral therapy (ART) efficacy. Long-term ART results in partial reconstitution of the immune system in people living with HIV-1 (PLWH) who start treatment in the chronic phase of infection, but the impact of ART initiation in the acute phase of infection is less studied. In this study, we investigate the effect of initiating ART in acute phase infection on the production of anti-Tat antibodies and on T-cell activation.

**Methods:**

Anti-Tat IgA, IgG, and IgM titres were evaluated longitudinally by enzyme-linked immunosorbent assay in plasma samples collected from 34 women who started ART immediately following the detection of acute HIV-1 infection. Total HIV-1 DNA measurements were performed by droplet digital PCR from total peripheral blood mononuclear cells at 1-year post ART initiation. T-cell activation was assessed longitudinally by analysis of the expression of HLA-DR and CD38 on CD4+ and CD8+ T-cells using flow cytometry. We also explored the association between anti-Tat antibody titres and CD4+ T-cell counts.

**Results:**

The data showed that anti-Tat IgG and IgM titres had decreased significantly after 12 months of treatment (p=0.0001) with no correlation between anti-Tat IgA, IgG or IgM and CD4+ T-cell counts (r= -0.09 to 0.2, p>0.05). There was no correlation between anti-Tat antibody levels and total HIV-1 DNA levels at ART initiation (r= 0.2143, p= 0. 6191) or after 12 months post-ART (r= -0. 2857, p= 0, 5008). There was a significant decrease in CD8+ T-cell activation between the baseline (day 1 on ART) and 12 months post-ART (p=0.0129).

**Discussion and conclusion:**

These findings suggest early initiation of ART reduces the production of anti-Tat antibodies and reduces CD8+ T-cell activation. Further studies on the impact of early ART on antiviral immune responses are needed and may shed light on mechanisms of optimal immune reconstitution and reservoir control in PLWH.

## 1 Introduction

The introduction of antiretroviral therapy (ART) has reduced the impact of HIV-1 infection into a manageable chronic condition. ART effectively decreases viral loads to undetectable levels, delays disease progression and restores T-cell immune function. ART also reduces the overall socioeconomic burden of HIV-1 by decreasing comorbidities and mortality rate of people living with the virus, and by preventing transmission to uninfected individuals (1, 2). However, the inability of ART to cure infection, the development of drug resistance and residual drug toxicity or side effects pose challenges to the ART success story. A vaccine to prevent new infections would be the most cost-effective approach to limit the spread of HIV-1, however, an effective vaccine remains elusive primarily due to the extreme genetic variation of the virus. Moreover, complete eradication of the virus in PLWH is also a significant scientific challenge with the major barrier being the presence of viral reservoirs of latently infected cells (3–5). These latently infected cells are not recognized by the immune system, and ART cannot eliminate these cells (6). To date, there is neither an effective preventative HIV-1 vaccine nor a cure for HIV-1, and therefore, there is a need to continue to search for innovative approaches to achieve these important goals.

The HIV-1 regulatory protein known as Tat increases viral transcription and plays an important role in disease progression. The major function of Tat is to stimulate transcriptional elongation after initiation of the process (7, 8). Tat is also essential in controlling latency and viral rebound following the interruption of ART (9). Evidence indicates that, when present in sufficient quantities, Tat may counteract the establishment of HIV-1 latency by promoting transcriptional initiation or elongation (10, 11). Tat protein is released into the extracellular space and into neighboring uninfected cells even without active HIV-1 replication and viral production, such as during effective ART (12). Extracellular Tat binds to trimeric Env on HIV-1, promoting engagement of arginine-glycine-aspartic acid (RGD) binding integrins, expressed by inflammatory dendritic cells (DCs), macrophages and endothelial cells (ECs) present at the site of infection. As a result, virions escape neutralization by anti-Env antibodies and enter target cells upon binding to RGD-binding integrins. Anti-Tat antibodies neutralize this binding, preventing virus entry through RGD-binding integrins (13).

Novel strategies are needed to enhance the effectiveness of ART, as it only partially restores immune functions and does not decrease the latent HIV reservoir. A therapeutic Tat vaccine strategy, has generated hope due to its encouraging outcomes in boosting ART, enhancing immune restoration, raising CD4+ T-cell count, and decreasing virus reservoirs more successfully than ART alone (14). Research points to the Tat vaccine as a viable vaccine option for strategies aimed at enhancing ART, depleting the HIV reservoir, and eradicating HIV-1 (14, 15). Previous research on HIV-1 infection has shown that the presence of an immune response specific to Tat is correlated with a lower incidence and a lower risk of developing AIDS in comparison to anti-Tat-negative individuals. This suggests that an immune response to Tat may play a protective role and regulate the *in vivo* progression to AIDS (16, 17). A study a combined Tat-based vaccine (Tat Oyi) and ART showed some promise as a strategy to control the reservoir of HIV-1 infected cells (18). Anti-Tat immunity, may also counteract Tat-mediated immunological dysregulation, thus significantly regulating HIV-1 disease and co-morbidity development (16, 19).

A previous study examined the relationship between various anti-Tat antibody isotypes and disease progression markers (plasma viral loads, CD4+ T-cell counts and T-cell phenotypes) in individuals with chronic non-B clade HIV infection (19). The study found that anti-Tat IgG alone was not protective unless it was present in combination with IgM, suggesting that persistent anti-Tat IgM has a protective role independent (19). Nevertheless, not enough research has been done on anti-Tat antibodies and how they may change, particularly in early treated HIV-1 infections. In the present study, we examine the evolution in anti-Tat IgA, IgG, and IgM from HIV-1 clade C infected women who initiated ART in acute phase of infection and were longitudinally followed for up to 12 months. Thereafter, we explored potential association between anti-Tat antibodies and total cellular HIV-1 DNA, CD4+ T cell counts and T-cell activation markers. Participants in our study were from the study measured the total anti-Tat-specific antibody titers in early-treated individuals in the Females Rising through Education, Support and Health (FRESH) cohort in Durban South Africa, which use a combination of a socioeconomic empowerment program and regular HIV-1 RNA screening to identify acute infection, with ART initiated immediately for those detected with acute HIV-1 infection (20, 21). Hence the study also examined the evolution of HIV-1 anti-Tat antibodies and markers of T-cell activation in women treated with ART from acute infection and up to 12 months post-ART.

## 2 Materials and methods

### 2.1 Study participants

#### 2.1.1 Females rising through education, support and health cohort

**Figure 1:**
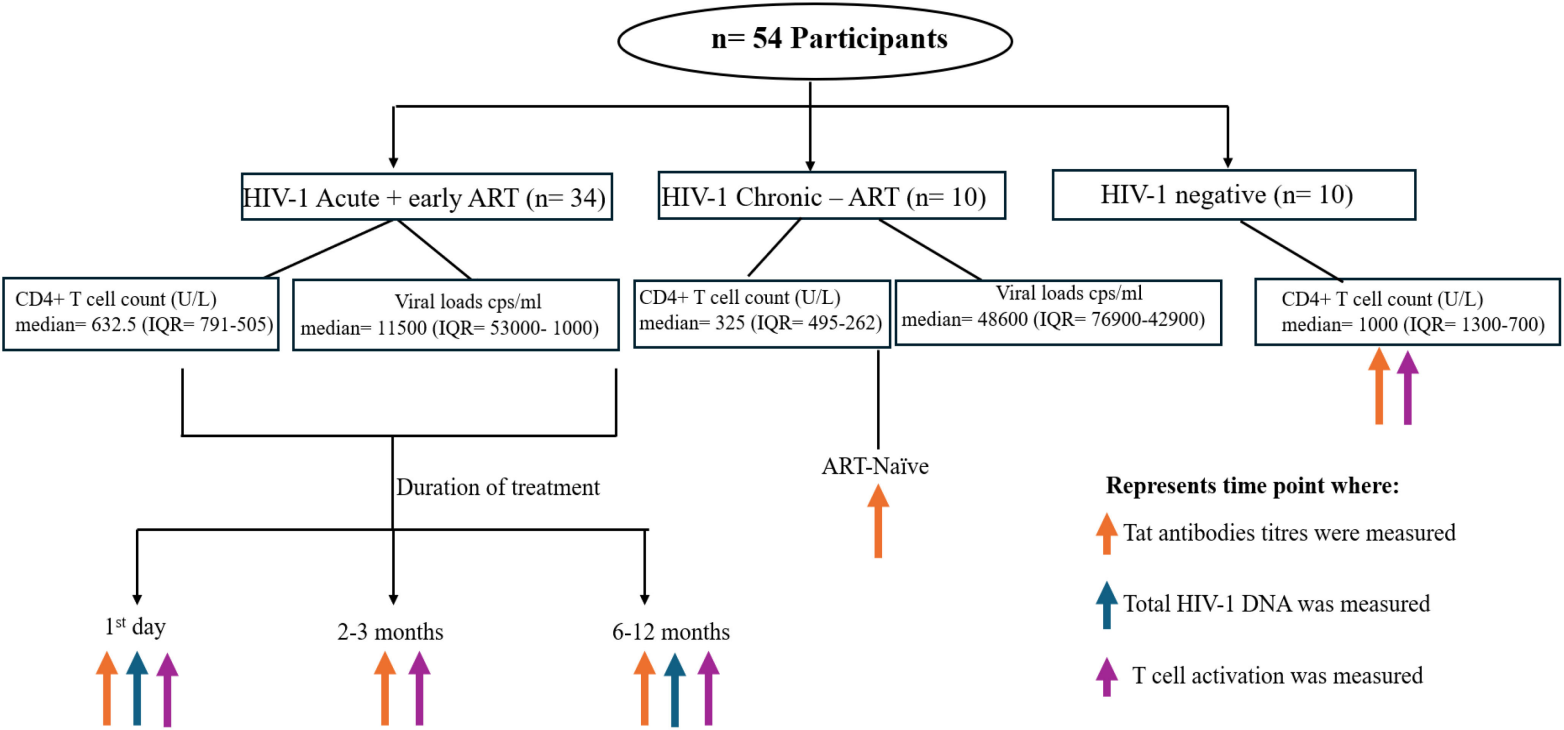
There were 34 acute treated participants analyzed, with samples collected on the day of ART initiation (day 1) and at 3 and 12 months post-ART. The controls were HIV-1 chronically infected ART naïve participants (n=10) and HIV-1 uninfected participants (n=10). Samples were obtained once for the latter 2 groups. The various assays performed at each timepoint are shown by the orange (anti-Tat antibodies), green (total HIV-1 DNA) and purple arrows (markers of T-cell activation).

The Females Rising through Education, Support and Health (FRESH) is an ongoing cohort study conducted in KwaZulu-Natal, South Africa. The goals of the FRESH study are to combine a socioeconomic interventional with basic science research (20, 21). Eligible women were HIV uninfected, aged 18–23 years, sexually active, not pregnant, non-anemic (hemoglobin ≥10 g/L), without other barriers to participation (serious chronic illness, enrolment in another study, or family responsibilities). Participants are screened for acute HIV-1 infection through twice weekly finger prick assessment for presence of plasma HIV-1 RNA. In the present study, human plasma samples were analyzed from 34 ART-treated women, who initiated treatment one day after detection of HIV-1 plasma RNA. **Table 1** shows the characteristics of the participants included in the study, and **Figure 1** is a summary of the study design.

**Table 1:** Characteristics of the HIV-1 participants included in the study.

Characteristics	Early treated^b^ (n= 34)	Chronic untreated^c^ (n= 10)	HIV-1 negative (n=10)
Median CD4 T cell counts ^a^ cells/μL	632.5 (791- 505)	325 (495- 262)	1000 (1300-700)
Median viral loads^a^ HIV-1 RNA copies/ml	11500 (53,000- 1,000)	48,600 (76,900- 42,900)	**-**

a Median values are shown with interquartile ranges in brackets.

b Early treated participants started ART 1 day following first detection of plasma viraemia.

c Chronic untreated participants were HIV-1 immunoassay seropositive participants who were ART-naïve.

The Biomedical Research Ethics Committee of the University of KwaZulu-Natal approved the study (BREC/00004500/2022). Participants gave written informed consent.

#### 2.1.2 Study participants

#### 2.1.3 Study design

Participants included in the present study were 34 women aged 18–23 years in KwaZulu-Natal, South Africa, who were HIV uninfected and sexually active consented to HIV-1 RNA testing twice a week. Participants were diagnosed with acute HIV-1 infection of whom were diagnosed in Fiebig stage I infection with a median initial viral load of 11500 copies/mL (IQR= 53000-1000). These participants started ART at a median of 1 day (1–1) after detection of plasma viraemia, which leads to a suppressed viral load (to <20 copies/mL) within 3 months of treatment.

### 2.2 Enzyme-linked immunosorbent assay

Human anti-Tat IgG, IgM and IgA were measured by ELISA in plasma samples collected from the participants. Ninety-six well Nunc™ MaxiSorp™ ELISA plates (BioLegend, San Diego, CA, US) were coated with 100 μL/well of HIV-1 clade C Tat (Sigma-Aldrich, St louis, MO,US) resuspended in phosphate buffered saline (PBS) (Gibco, New York, US), overnight at 4°C. Plates were then washed three times with PBS containing 0.05% Tween-20 (Sigma-Aldrich, St louis, MO, US) and then blocked with PBS containing 5% BSA (Thermo Fisher, Waltham, MA, US) for 2 hours at room temperature. Plates were washed three times, and 100 μL/well of appropriate dilutions of each plasma diluted in PBS were dispensed in duplicate wells and incubated for 2 hours at room temperature. Plates were washed three times before the addition of 100 μL/well of HRP-conjugated anti-human IgG (Sigma-Aldrich, St louis, MO, US), HRP-conjugated anti-human IgA (Sigma Aldrich, St louis, MO, US), or HRP-conjugated anti-human IgM (Sigma Aldrich, St louis, MO, US) diluted 1:1, 1:10 and 1:100, in PBS and incubated for 1 hour at room temperature. After incubation, plates were washed three times and 50μL of the substrate (1 o-phenylenediamine dihydrochloride (OPD) tablet (Thermo Fisher, Waltham, MA, US), 11mL of phosphate citrate buffer (Sigma Aldrich, St louis, MO, US) and 4.4 μL H_2_O_2_ (ReAgent, Runcorn, Cheshire, UK)) was added for detection. 50 μL of H_2_SO_4_ was added to stop the reaction. Absorbance in optical density was measured at 490 nm using a Victor Nivo multimode plate reader (PerkinElmer, Waltham, MA, US). Plasma from HIV-1-negative participants were used as negative controls, and plasma from ART-naïve participants with chronic HIV-1 infection were used as positive controls. The cut-off value was estimated as the mean of the negative controls. A plasma sample with a higher value than the cut-off value was considered positive for anti-Tat antibodies. GraphPad Prism was used, titers were calculated using different dilutions to get the area under the curve.

### 2.3 Measurement of the total HIV-1 DNA by droplet digital PCR

Total peripheral blood mononuclear cells (PBMCs) collected from each participant at each time point were independently subjected to DNA extraction using DNeasy Blood & Tissue Kits (QIAGEN, Germantown, Maryland, US). Total HIV-1 DNA and host cell concentrations in the DNA extracts were estimated using Bio-Rad droplet digital PCR (ddPCR), using primers and probes covering HIV-1 5′ LTR-gag HXB2 coordinates 684–81037 (forward primer 5′-TCTCGACGCAGGACTCG-3′, reverse primer 5′-TACTGA CGCTCTCGCACC-3′ probe/56-FAM/CTCTCTCCT/ZEN/TCTAGCCTC/31ABkFQ/, and human RPP30 gene forward primer 5′-GATTTGGACCTGCGAGCG-3′, reverse primer 5′-GCGGCTGTCTCCACAAGT-3′, probe/56 FAM/CTGACCTGA/ZEN/AGGCTCT/31ABkFQ/). ddPCR was performed using the following thermocycler program: 95°C for 10 min, 45 cycles of 94°C for 30 s, and 60°C for 1 min, and 72°C for 1 min. The Bio-Rad QX100 droplet reader (Bio-Rad, Hercules, CA, US) subsequently read the droplets and data were analyzed using QuantaSoft software (Bio-Rad) edition 1.2.

### 2.4 Calculations of area under the curve for viral load

The area under the curve (AUC) for viral load in participants longitudinally followed from ART initiation in acute HIV-1 infection was calculated to quantify overall exposure to the virus before full viral suppression. The AUC was determined by integrating viral load measurements taken from the time of first HIV-1 viral RNA detection to the day the participant’s viral load dropped below 20 copies/mL (undetectable viral load). A graph was plotted with days post onset of plasma viraemia (DPOPV) on the X-axis and viral load (log copies/mL) on the Y-axis. The AUC was then calculated using GraphPad Prism version 5. This method provides a comprehensive summary of viral load dynamics, offering insights into how viral load exposure may correlate with disease progression and the efficacy of ART.

### 2.5 Measurement of immune activation by flow cytometry

PBMCs from participants were isolated by Ficoll density gradient centrifugation and cryopreserved until use. Cryopreserved samples were thawed and resuspended in R10 medium (RPMI (Sigma- Aldrich, St louis, MO, US), 5.5 mL L-glutamine (Gibco, Grand Island, NY, US), 5.5 mL Penicillin- Streptomycin (Thermo Fisher, SA), 5.5 mL Hepes (Thermo Fisher, Waltham, MA, US), 10% FBS (Gibco, Grand Island, NY, US). Cells were rested for 2 hours at 37 ^0^C before staining. For immunophenotyping one million cells were stained and incubated for 20 minutes using the following fluorochrome-labelled monoclonal antibodies (MAbs): CD3-BV650/BV786, CD4-APC, CD8-FITC, CD56-BV510, CD38-BV711, HLA-DR-PECF594 (BD Biosciences, San Diego, CA, US) and fixable viability dye (Thermo Fisher, Waltham, MA, US), before the 20 minutes fixation with Fix and Perm Medium A (Thermo Fisher, Waltham, MA, US). Acquisition was performed on an LSR Fortessa (BD Biosciences, San Diego, CA, US). Compensation was conducted with antibody capture beads (BD Biosciences, San Diego, CA, US) stained separately with the individual antibodies used for sample staining. Flow cytometry data was analyzed using FlowJo version 10.8.1. The gating strategy for T-cell activation assessment is shown in **Supplementary Figure 1**. Briefly, single and viable cells were gated from the total lymphocytes to omit doublets and non-viable cells from the analyses. Following their identification, CD3+ T-cell lymphocytes were gated for CD4+ and CD8+ T-cell subsets and activation was defined by HLA-DR and CD38 expression.

### 2.6 Software and statistical analysis

FlowJo software, version 10.81, was used for flow cytometry data analysis. Data analyses were performed using Graphpad Prism version 5 (GraphPad Inc.) for graphical display and Microsoft Excel (Microsoft). Groups were compared using the Mann-Whitney U-test. For association analyses, the Spearman rank correlation was determined. P-values ≤ 0.05 were regarded as statistically significant.

## 3 Results

### 3.1 Longitudinal analysis of anti-Tat antibodies in HIV-1 early treated individuals

**Figure 2:**
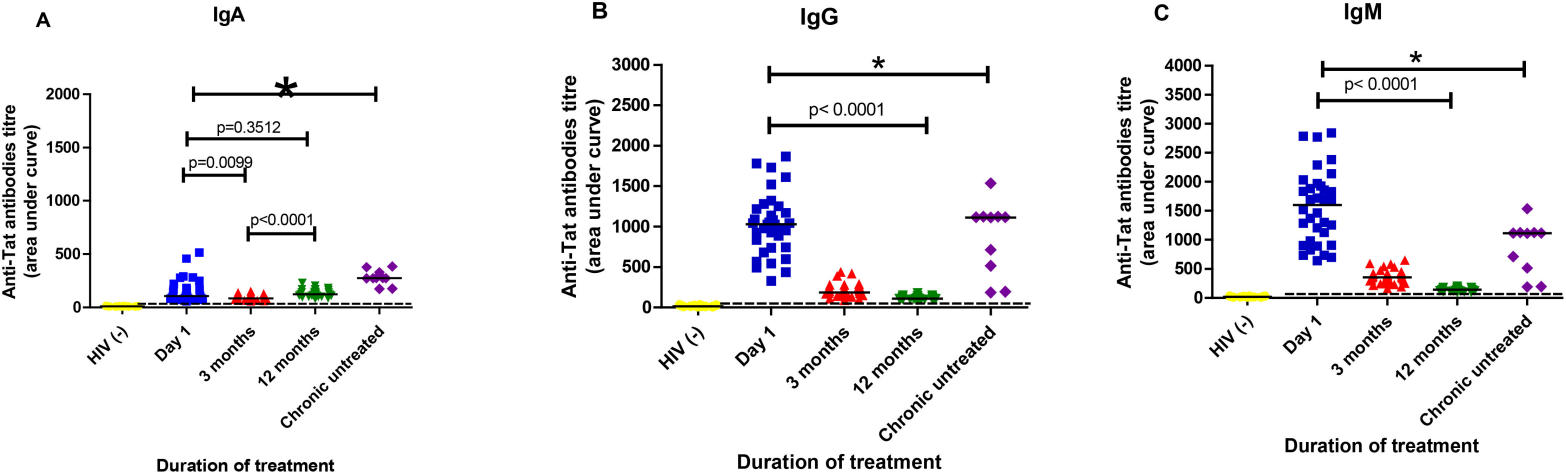
Longitudinal analyses of anti-Tat antibody isotypes in PLWH who initiated ART during acute infection, with HIV-1 negative and chronically infected ART-naïve samples as negative and positive controls respectively. Antibody isotypes compared were **(A)** IgA, **(B)** IgG and **(C)** IgM. Y-axes represent anti-Tat antibody titers, and X-axes represent different post-ART time points. Highlighted in yellow are HIV-1 uninfected negative controls, followed by acute treated participants at day 1 (blue), 3 months (red) and 12 months (green) post-ART and chronically infected ART-naïve participants (purple). The straight line within the data points represents the median value. Statistical comparisons were made using the Mann-Whitney test. * represent the significant p value.

Plasma samples from early treated HIV-1 individuals were first tested for anti-Tat IgA, IgG and IgM antibodies recognizing clade C Tat (**Figure 2**). **Figure 2** shows the anti-Tat antibody titers for different isotypes within the first year of ART initiation in early treated individuals. HIV-1 chronically infected ART-naïve participants were included as positive controls, and the cut-off value was estimated as the median of the negative controls (HIV-1 uninfected participants). The median for anti-Tat IgA titer detected on day 1 of ART was 106.7 Ab titer; it was 84.6 Ab titer at 3 months post-ART, and it was 124.3 Ab titer at 12 months post-ART (**Figure 2A**). The median for anti-Tat IgG titers were 1029, 185.6, and 110.8 at day 1, 3 months and 12 months post-ART, respectively (**Figure 2B**). The median for anti-Tat IgM titers were 1601, 356.3, and 140.9 at day 1, 3 months and 12 months post-ART, respectively (**Figure 2C**). All individuals had higher anti-Tat antibodies than the Ab titer cut-off values of 10.53, 19.61, and 19,61 for IgA, IgG, and IgM, respectively. These results indicate that anti-Tat IgG and IgM titers had decreased significantly after 12 months of treatment (p<0.0001). There was no statistical difference detected between day 1 and 12 months post-ART for anti-Tat IgA titers (p= 0.3512). However, there was a statistical difference when compared day 1 and 3 months post-ART treatment (p=0.0099), and between 3 and 12 months post-ART treatment (p<0.0001). Anti-Tat IgA and IgG antibodies in HIV-1 chronically infected ART-naïve participants were significantly higher compared to early treated participants (p<0.05). However anti-Tat IgM in day 1 was significantly higher compared to HIV-1 chronically infected ART-naïve participants (p< 0.05). These analyses also demonstrate that a large proportion of the 34 HIV early HIV- treated individuals had plasma anti-Tat antibody responses dominated by IgG and/or IgM very early in infection.

### 3.2 Correlation between anti-Tat antibodies and CD4+ T-cell count and viral load

Anti-Tat antibodies have been shown to correlate with markers of disease progression such as CD4+ T-cell count and viral loads in chronic HIV-1 infection (19). Here, we investigated if there was correlation between anti-Tat antibody isotype levels and CD4+ T-cell count in HIV-1 early treated participants to understand how the immune system responds to HIV-1 and is impacted by treatment. Thirty-four plasma samples of HIV-1 early treated individuals from different time points were selected according to their duration of treatment. **Supplementary Figures 2A-C** showed that there was no significant correlation (r= -0.02 to 0.2, p>0.05) between anti-Tat antibody levels measured at day 1 of treatment and CD4+ T-cell counts at day 1, 3 months or 12 months post-ART. Additionally, correlation between ant-Tat antibody levels and viral load on day 1 of treatment was analyzed, with no correlation observed (r= -0.25 to 0.17, p> 0.05) (**Supplementary Figure 2D**). Following the initiation of ART all participants maintained undetectable viral loads (<20 copies/ml) at 3- and 12-months post-ART. We also analyzed for correlation between anti-Tat antibody isotypes and CD4+ T-cell count and viral load in the HIV-1 chronically infected ART-naïve individuals. **Supplementary Figure 3A** shows that there was no correlation between any of the anti-Tat antibody isotype titers and CD4+ T cell count (r= 0.05 to 0.50, p> 0.05). **Supplementary Figure 3B** shows that there was no significant correlation between anti-Tat antibody titers and viral load (r= -0.7052 to 0.1094, p> 0.05), except for IgA where the isotype levels negatively correlated with viral load (r= -0.7052, p= 0.0268). For further analyses, correlation between anti-Tat antibodies titer and area under curve of viral load in HIV-1 early treated participants. **Supplementary Figure 4** showed how the area under curve of the participants looked like. It was observed that the viral load was suppressed overtime. **Supplementary Figures 5**-**7** suggest that there is no correlation between the level of anti-Tat antibodies and virus attack on the participants (r < 1, p >0.05).

### 3.3 Correlation between anti-Tat antibodies and total HIV-1 DNA

We further analyzed the possible relationship between the level of anti-Tat antibody titers and the total HIV-1 DNA after 12 months post-treatment. Total HIV-1 DNA (Log copies/10^6^ cells) was measured from 8 plasma samples that showed the presence of anti-Tat antibodies in all the isotypes on day 1 and 12 months of treatment using ddPCR. There was no correlation between the level of anti-Tat antibodies and total HIV-1 DNA (r= 0.2, p >0.05) (Figure not shown). However, the detection of anti-Tat antibodies, as well as the total HIV-1 DNA, decreases with prolonged therapy (p< 0.05) (**Supplementary Figure 8**). We further analyzed for correlation between total HIV-1 DNA and viral load area under the curve was analyzed. **Supplementary Figure 9** suggests no correlation between the total HIV-1 DNA and area under the curve of the viral load (r< 1, p >0.05).

### 3.4 Association between anti-Tat antibodies and T-cell activation

**Figure 3:**
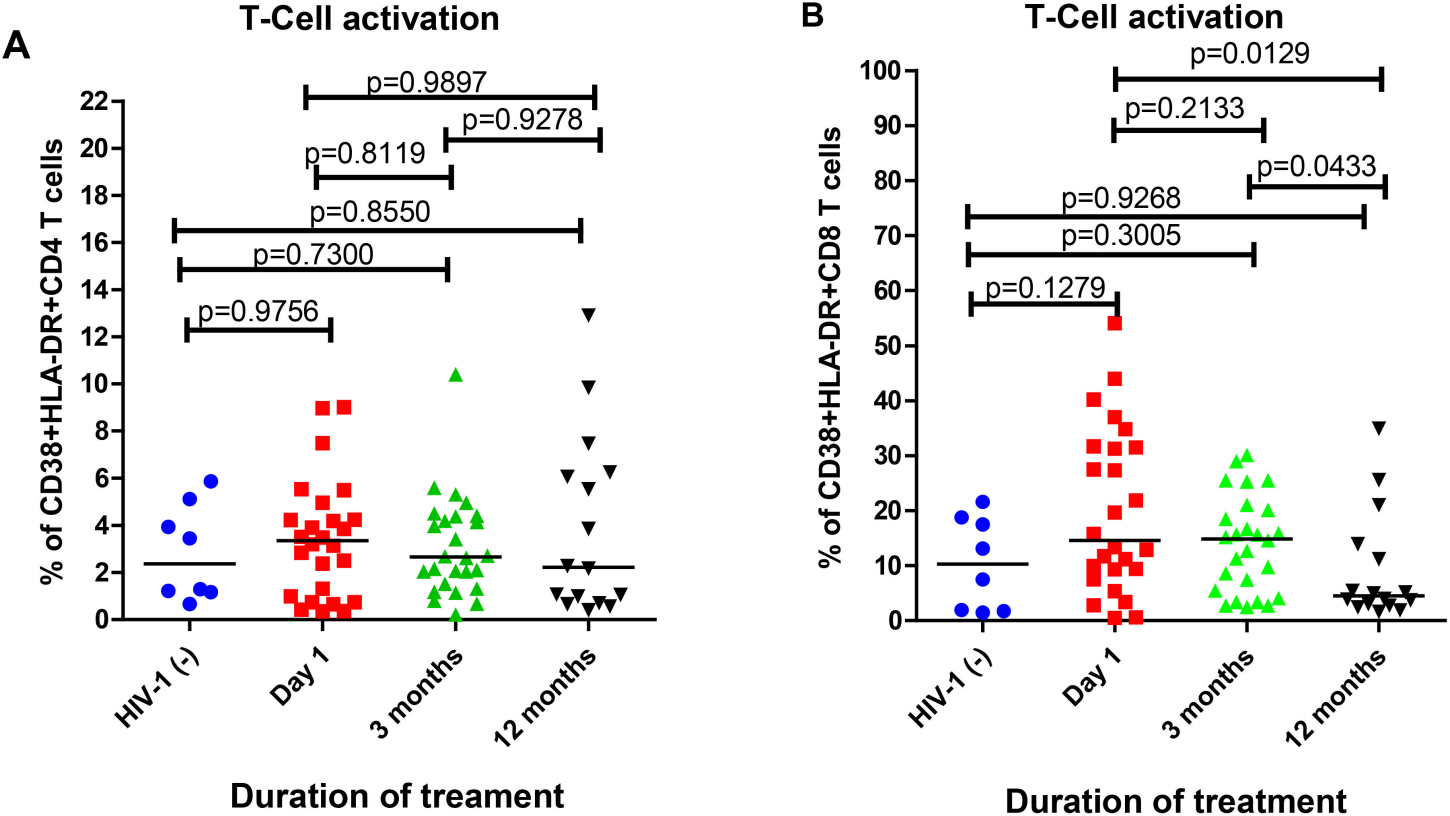
T-cell activation frequencies in HIV-1 uninfected controls and in participants who initiated ART in acute HIV-1 infection. **(A)** The percentages of CD4+ T-cells expressing CD38 and HLA-DR and **(B)** The percentages of CD8+ T-cells expressing CD38 and HLA-DR. The T-cell activation frequencies are shown on the Y-axes, while the treatment duration is shown by X-axes. Shapes highlighted in blue, red, green and black represent HIV-1 negative, day 1, 3 months and 12 months of infection, respectively. Statistical comparisons were performed using the Mann-Whitney test.

HIV-1 disease progression is characterized by T-cell abnormalities such as persistent activation. Activation profiles of CD4+ and CD8+ T-cells by assessing CD38 and HLA-DR co-expression was evaluated, as shown in **Figure 3**. **Figure 3A** shows that there were no statistical differences in CD4+ T-cell activation between the time points analyzed within the first year of treatment (p>0.05). Similar frequencies of CD4+ T-cell activation were observed at 3 and 12 months compared to HIV-1 negative participants; no statistical differences were observed (p= 0.7300; p= 0.8550, respectively). As shown in **Figure 3B**, acutely treated participants had similar levels of CD8+ T-cell activation on day 1 of treatment compared to HIV-1 negative participants, however no statistical difference was observed (p= 0.1279). Following the initiation of therapy, CD8+ T-cell activation was reduced such that the frequency of activated CD8+ T-cells at 12 months was lower compared to those on day 1 of treatment (p=0.0129), although this was not statistically different from the frequency of activated CD8+ T-cells in HIV negative participants (p= 0.1279). There was no correlation between anti-Tat antibody titers and T-cell activation over time across all isotypes (-1< r >1, p > 0.05) (**Supplementary Figure 10**). Overall, these results indicate that reduced CD8^+^ T-cell activation was more closely associated with early ART initiation rather than with the presence or titer of anti-Tat antibodies, resulting in levels of CD8+ T-cell activation that are comparable to HIV-1 negative individuals. No statistically significant correlation was observed between anti-Tat antibody levels and T-cell activation markers.

## 4 Discussion

Antiretroviral therapy (ART) suppresses HIV-1 replication to levels that are undetectable in the peripheral blood with the lower limit of detection being 20 or 50 copies/ml of HIV-1 RNA depending on the assay. However, ART does not eradicate the virus, even after decades of treatment. Tat-specific antibodies have been found to be associated with lower disease progression in untreated patients (19). In this study, the longitudinal evolution of Tat-specific antibodies from early treated PLWH was determined from the first day to 12 months of infection. Consistent with Keating et al. (22) and Nicoli et al. (19), the present study shows that initiation of ART significantly reduces anti-Tat clade C antibodies (IgG and IgM). It is interesting that in this cohort of acute treated HIV-1 infection, most participants had already developed anti-Tat antibodies even though they did not display full antibody seroconversion as previously described (20). However, there was no correlation between anti-Tat antibody isotypes and markers of disease progression except for anti-Tat IgA which showed a negative correlation with viral load (**Supplementary Figure 3B**). Moreover, there was no correlation between the levels of anti-Tat antibodies and total HIV-1 DNA at 12 months post ART, suggesting that these antibodies did not contribute to reservoir reduction. However, this result needs to be interpreted with caution due to the small sample size of our study. ART initiated in acute infection also reduced CD8+ T-cell activation over the 12 months of follow up.

Enzyme-linked immunoassay assay was performed to measure the presence of Tat clade C antibodies in the plasma of early treated participants and demonstrated that anti-Tat IgG and IgM decreased over time, with no significant difference observed in anti-Tat IgA titer between day 1 and 12 months post-ART. Whereas robust anti-Tat IgG and IgM titers were observed in these early treated participants (median AUC values of 1029 and 1601, respectively), Tat IgA levels were much lower at a median titer AUC of 106.7, likely a reflection of IgA being more robustly expressed in mucosal tissues. High responses of all anti-Tat antibodies in chronically HIV-1 ART-naive individuals were observed compared to HIV-1 early treated (**Figure 2**). Our results are consistent with the study by Keating et al. (22) who reported a progressive decline in antibody responses due to ART-induced viral suppression that lasted for 5–7 years. However, unlike Keating et al. (22), we did not observe a correlation between anti-Tat antibodies and viral load, likely because participants in our study started treatment in acute infection or the sample size was too small for the chronically infected ART-naïve participants.

The current study shows that IgG and IgM responses dominate in the early stages of treatment (**Figures 2B, C**). IgM (unswitched antibody) is the first antibody isotype produced during an immune response, followed by the class-switched antibodies, IgG and IgA. Moreover, IgM functions as a primary barrier against HIV-1 and regulates immune responses (23). IgM is highly efficient in activating the complement system and inhibiting virus entry by directly interacting with HIV-1 co-receptors (24). The persistence of IgM during chronic infection is interesting and has been recently described for other diseases (**Figure 1**) (25). In addition, Tat IgM has been observed to also persist in Tat-vaccinated subjects, suggesting that Tat-specific IgM+ memory B cells are long-lived (26). Individuals with detectable IgM and IgG antibodies responses displayed higher responses at the early stages of infection shortly after treatment initiation, which declined over time. According to these results, a lowering of anti-Tat antibody levels during early ART may reflect decreased viral production.

Interestingly, IgA responses reduced from the initiation of treatment to 3 months of infection and a recovery of responses at 12 months of infection was observed (**Figure 2A**). The role of serum HIV-specific IgA has been previously debated with some reports indicating that serum IgA may display neutralizing activity (19). Results from the RV144 trial demonstrated that serum anti-Env IgA may counteract the activity of protective IgG (27). HIV-infected individuals with Tat IgA showed significantly higher pVL and activation of CD8+ T-cells and lower CD4+ T-cell counts (19). There was no evidence of accelerated disease progression in these subjects, but firm conclusions are not possible participants in our study were initiated on ART during acute infection and the longitudinal follow-up was limited to one year.

Previous studies have shown a significant correlation between anti-Tat immunity and high CD4+ T-cell counts and control of low viral loads in people living with HIV-1 who are chronically untreated or on ART (19, 28). A subsequent study reported that anti-Tat IgM correlated with higher CD4+ T-cell counts and lower viral loads irrespective of the duration of chronic infection in ART-naive patients (19). In this study, we reported no correlation or statistical significance between anti-Tat antibodies and CD4+ T-cell count and viral load for both HIV-1 early treated and chronically HIV-1 ART-naïve participants (**Supplementary Figures 2**, **3**). However, there was significance between IgA and viral load in chronic group (**Supplementary Figure 3B**). This may be because most participants had relatively high (>500 copies/ml) CD4+ T-cell count and initiated treatment early. The viral loads of the present study HIV-1 early treated participants remained less than 20 copies/ml following the initiation of ART (20). In addition, no correlation was observed between anti-Tat antibodies and area under the curve of viral load in early-treated participants. Thus, we cannot conclude that anti-Tat immunity influenced the drastic decrease of the viral load in HIV-1 early treated participants.

HIV-1 Tat protein abnormalities have been found to have an impact on the establishment and maintenance of latent infection (3). Thus, we ascertained the disparity between anti-Tat antibodies and the total HIV-1 DNA. Acute infection creates the latent reservoir, which is primarily made up of CD4+ T cells with resting memory. Research indicates that Tat, in sufficient amounts, may be able to prevent HIV-1 latency from developing by promoting transcription initiation or elongation (10, 11). The current study found that, there was no correlation between the level of anti-Tat antibodies and the total HIV-1 DNA, not statistically significant (Figure not shown). Our results are consistent with a study by Reddy et al. (29), which found that although ART started during hyperacute HIV-1 subtype C infection did not affect reservoir seeding, it was linked to a faster decay of intact viral genomes. The current study found no correlation; however, we observed that total HIV-1 DNA was high during the acute phase of the infection than they were a year later, and they reduced after 12 months post-ART (**Supplementary Figure 8**). Because latent proviruses do not produce viral gene products, they are protected from both ART drugs and the host immune response. The long-lived, latently infected host cell remains unaffected by viral cytopathic effects (30). Hence, we can conclude that the decrease in total HIV-1 DNA maybe be associated with the earlier ART initiation (31).

This study demonstrates that in the early-treated PLWH, anti-Tat antibodies are linked to the decrease of CD4+ and CD8+ T-cell activation (**Figure 3**). Similarly, anti-Tat antibodies were linked to low T-cell activation in individuals with chronic infection (19). Consistently, we observed that patients with positive IgA, IgG and IgM responses on day 1 of infection displayed high frequencies of HLA-DR^+^CD38^+^ CD4^+^ T cells compared to HIV-1 negative individuals, but not significant. Almost similar frequencies of HLA-DR^+^CD38^+^ CD4^+^ T-cells were observed at 3 and 12 months compared with HIV-1 negative, but not significant (**Figure 3A**). According to Naidoo et al. (32), T-cell activation was measured at 1 month and 12 months for PLWH that initiated ART in the hyperacute stage of infection. The median T-cell activation was lower compared to HIV-1 negative individuals, but not significant (32). In our study, HLA-DR^+^CD38^+^ CD8+ T-cell activation significantly decreased with prolonged treatment (**Figure 3B**), this implies that early initiation of ART reduced virus associated immune activation (33). In addition, there was no correlation between the presence of anti-Tat antibodies and T-cell activation in HIV-1 early treated participants (**Supplementary Figure 10**). However, Nicoli et al. (19) reported that anti-Tat IgM were preferentially detected in chronic HIV-infected subjects with low T cell activation (*p-v*alue = 0.03). High CD8+ T-cell activation was observed compared to CD4+ T cell activation (**Figure 3**). CD8+ T-cells are critical in the natural and ART-induced control of viral replication; however, CD8+ T-cells are highly affected by the persistent immune activation and exhaustion state driven by the increased antigenic and inflammatory burden during HIV-1 infection, inducing phenotypic and functional alterations and hampering their antiviral response (34).

The limitations of the current study include a small sample size, lack of matching samples to analyze some of the parameters at every time point and a relatively short longitudinal follow up period. These limitations necessitate caution in our data interpretation and make it challenging to generalize our findings. Therefore, further experiments with a larger sample size is needed to explore the link between anti-Tat immunity, markers of disease progression and total HIV-1 DNA following early ART initiation. The current study could not compare T-cell activation of patients with and without anti-Tat antibodies because of the limited sample size. Therefore, future studies should have a long duration of longitudinal follow-up and increase the number of samples. In addition, determining a direct association between levels of anti-Tat antibodies and T-cell activation in HIV-1 participants is recommended for potential future studies. Nevertheless, longitudinal samples from early treated HIV-1 participants (FRESH cohort) allowed us to observe the evolution of anti-Tat antibodies, the association between anti-Tat antibodies and the total HIV-1 DNA, and immune activation.

In summary, early treatment reduces the production of anti-Tat antibodies as they gradually deteriorate, and they appear to have no impact on the total HIV-1 DNA decay. Anti-Tat antibody levels did not correlate with CD4+ T-cell counts. Early ART administered for up to 12 months, lowered CD8+ T-cell activation. These findings highlight the importance of early initiation ART in mitigating the detrimental effects of HIV-1 on the immune system, thereby improving long-term clinical outcomes and reducing the risk of immune dysfunction and progression to AIDS.

## Data Availability

The original contributions presented in the study are included in the article/**Supplementary Material**. Further inquiries can be directed to the corresponding author.
