# Supplementary material for: Antiretroviral therapy initiated during acute infection in women with HIV-1 clade C reduces anti-Tat antibody production and lowers CD8+ T cell activation
Source: Front Immunol. 2025 Jun 18;16:1564960. doi: 10.3389/fimmu.2025.1564960 (PMC12213416; doi:10.3389/fimmu.2025.1564960)
Supplement: Supplementary file 1 [file DataSheet1.pdf]

Supplementary Figures

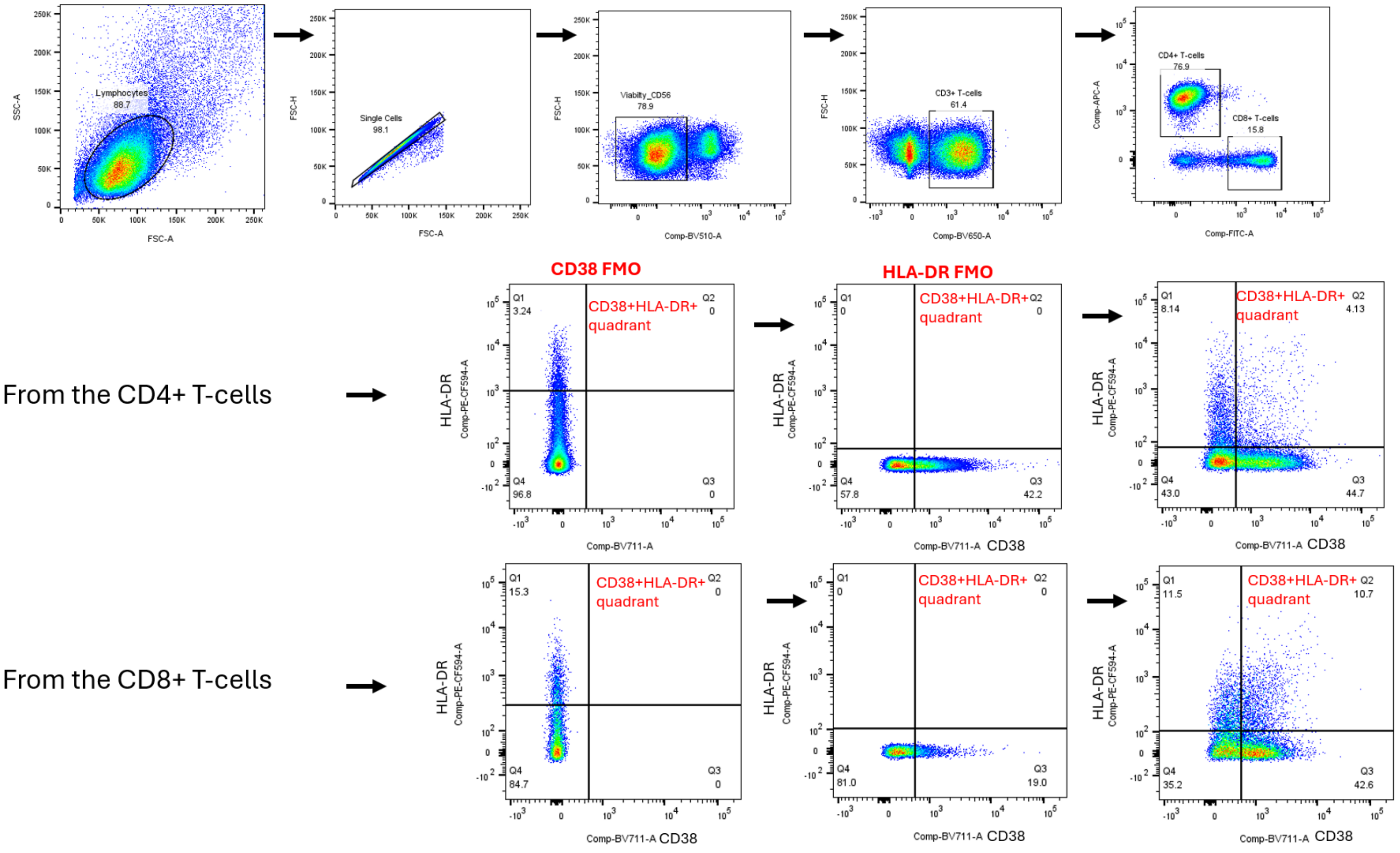

Figure 1: Gating strategy for assessing T cell activation markers: Frequencies of CD4+ and CD8+ T cells expressing activation markers in the PBMCs; Shown are representative plots demonstrating the gating strategy for the expression of activation (CD38 and HLA-DR). Samples were initially gated on lymphocytes based on SSC-A/FSC-A gating (A). Single cells were gated using FSC-H/FSC-A gating (B). Dead cells and other unwanted cells were excluded (C). T cells were gated as CD3+ cells (D). Subsets of T cells were further gated based on CD4 and CD8 expression (E). T cell activation was determined using activation markers; HLA-DR$^+$ CD38$^+$ on CD4$^+$ and CD8$^+$ T cells (F). The background was removed for FMOs. The time gate was not used.

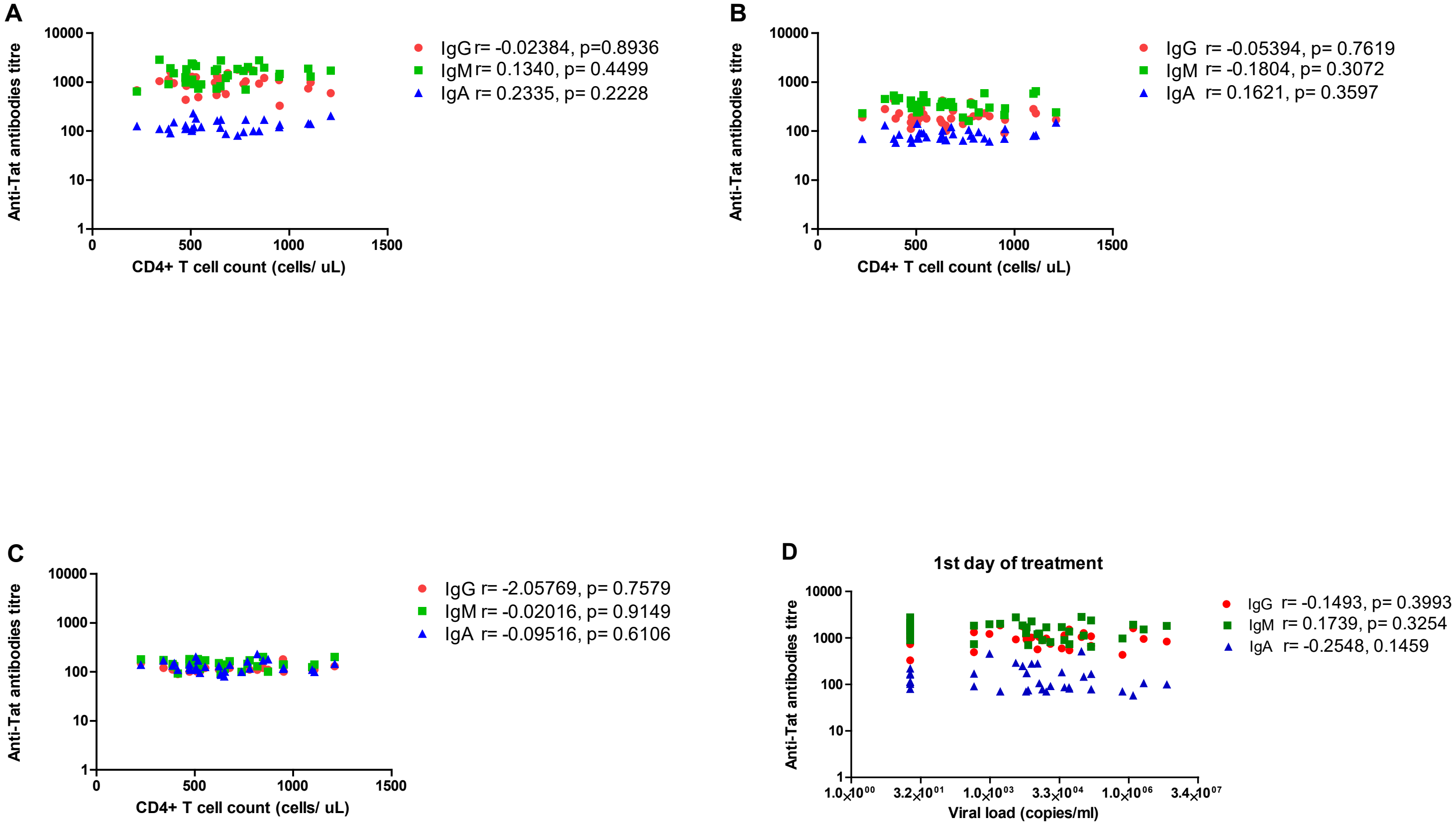

Figure 2: Correlation between anti-Tat antibodies isotypes of HIV-1 early-treated individuals and disease progression markers. Subgroups were compared between different anti-Tat antibody isotypes at (A) Day 1, (B) 3 months, (C) 12 months of treatment and (D) Viral load on day 1 of treatment. Y-axes represent Anti-Tat

Antibody titres, and X-axes represent CD4+ T cell count (cells/ul) and Viral load (copies/ml). Shapes highlighted in blue, red, and green represent IgA, IgG, and IgM, respectively. Statistical comparisons were made using the spearman test.

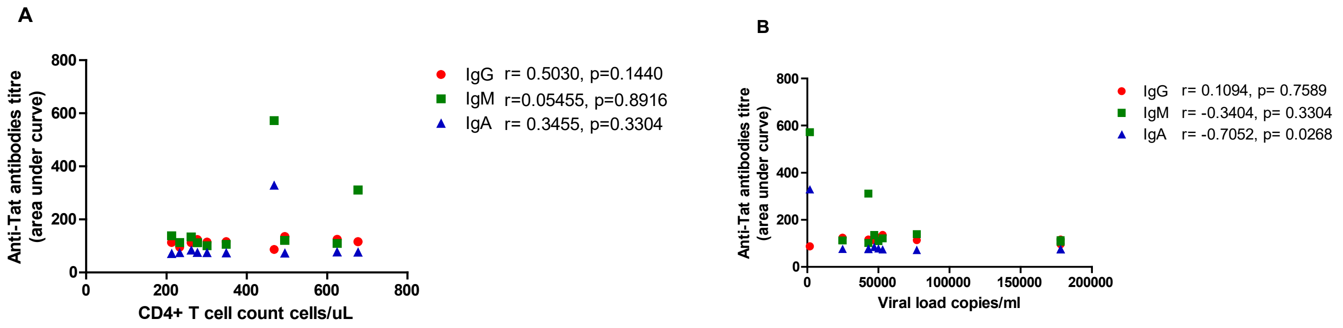

Figure 3: Correlation between anti-Tat antibodies isotypes and CD4+ T cell count and viral load of chronically HIV-1 ART-naïve individuals. Subgroups were compared between different anti-Tat antibody isotypes at chronic stage of infection. Y-axes represent Anti-Tat Antibody titres, and X-axes represent CD4+ T cell count (U/L) and viral load (copies/ml). Shapes highlighted in blue, red, and green represent IgA, IgG, and IgM, respectively. Statistical comparisons were made using the spearman test.

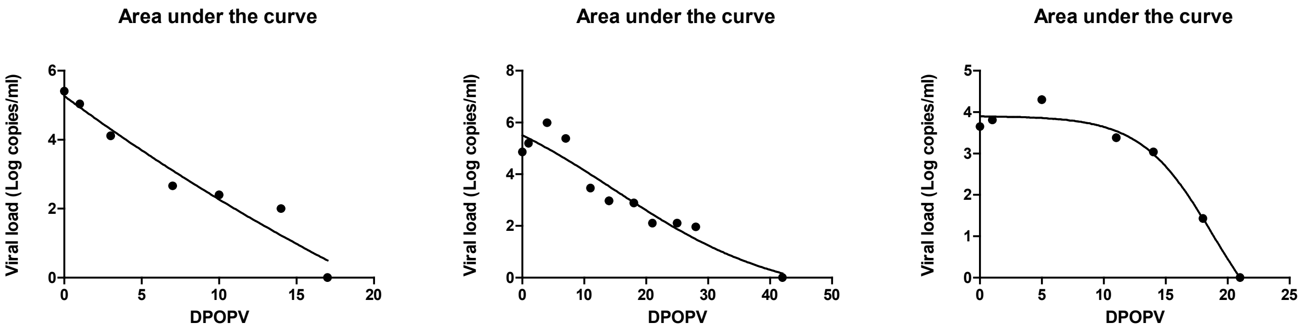

Figure 4: Area under the curve of viral load. Y-axes represent viral load (log copies/ml) and x-axes represent Days of Post Plasma Viraemia. Nonlinear regression was used for curve fitting method.

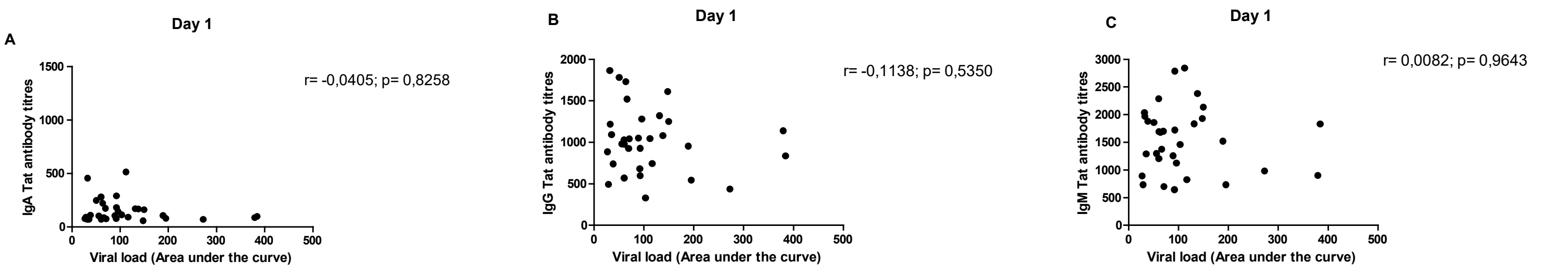

Figure 5: Correlation between anti-Tat antibodies and viral load (area under the curve). Subgroups were compared for (A) IgA, (B) IgG and (C) IgM. Y-axes represent anti-Tat antibody titres and x-axes represent viral load (area under the curve). Statistical comparisons were made using the Spearman (Nonparametric correlation).

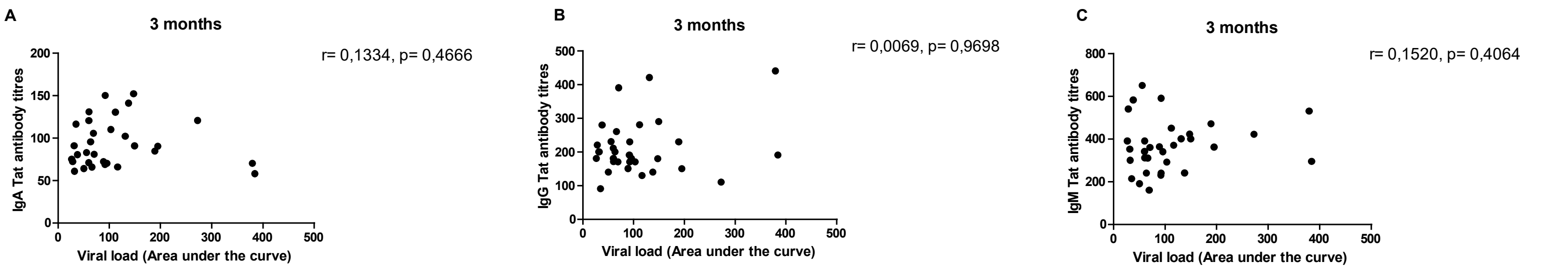

Figure 6: Correlation between anti-Tat antibodies and viral load (area under the curve). Subgroups were compared for (A) IgA, (B) IgG and (C) IgM. Y-axes represent anti-Tat antibody titres and X-axes represent viral load (area under the curve). Statistical comparisons were made using the Spearman (Nonparametric correlation).

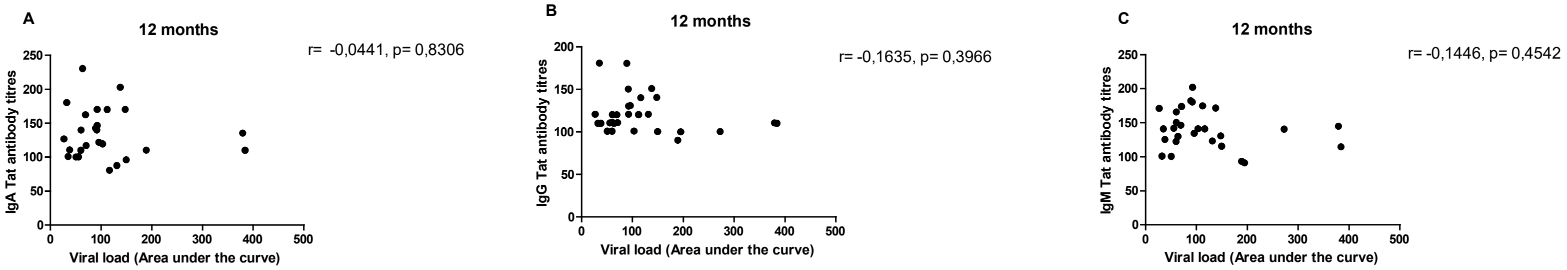

Figure 7: Correlation between anti-Tat antibodies and viral load (area under the curve). Subgroups were compared for (A) IgA, (B) IgG and (C) IgM. Y-axes represent anti-Tat antibody titres and X-axes represent viral load (area under the curve). Statistical comparisons were made using the Spearman (Nonparametric correlation).

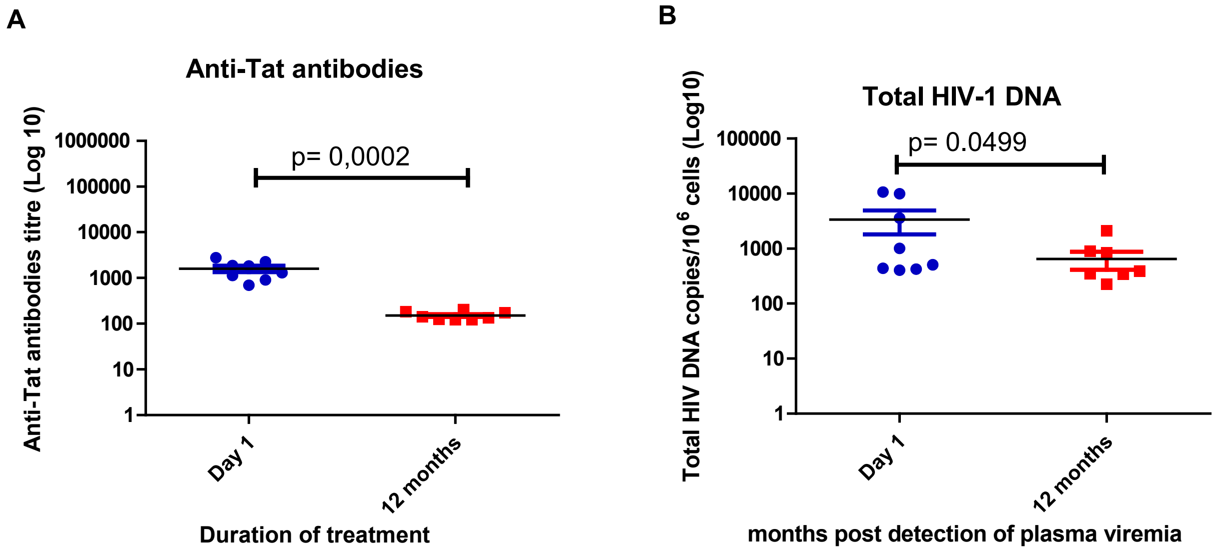

Figure 8: Tat antibodies as well as the total HIV DNA, decreases with prolonged therapy. Subgroups were compared for day 1 (blue) and 12 months (red) of treatment. Y-axes represent (A) Tat antibody titres, (B) Total HIV-1 DNA and X-axes represent duration of treatment. Statistical comparisons were made using the Spearman (Nonparametric correlation).

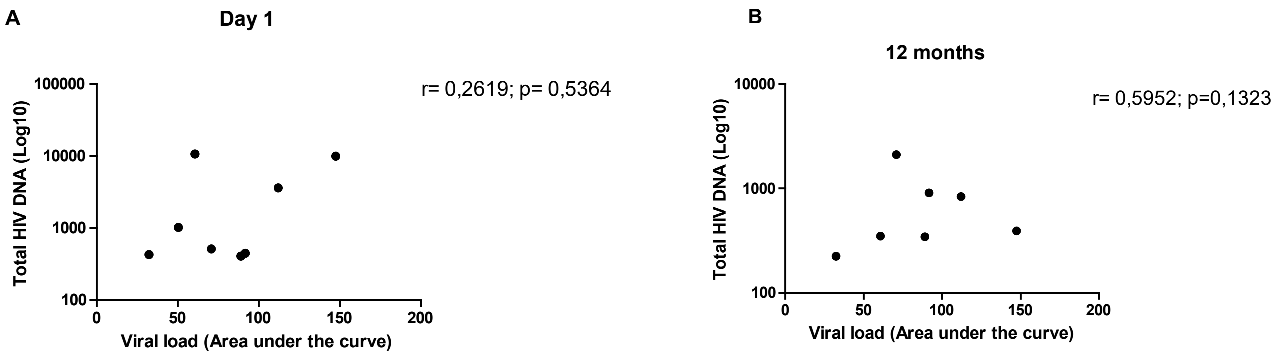

Figure 9: Correlation between Total HIV DNA and viral load (area under the curve). Subgroups were compared for (A) day 1 of treatment and (B) 12 months of treatment. Y-axes represent Total HIV DNA, and x-axes represent viral load (area under the curve). Statistical comparisons were made using the Spearman (Nonparametric correlation).

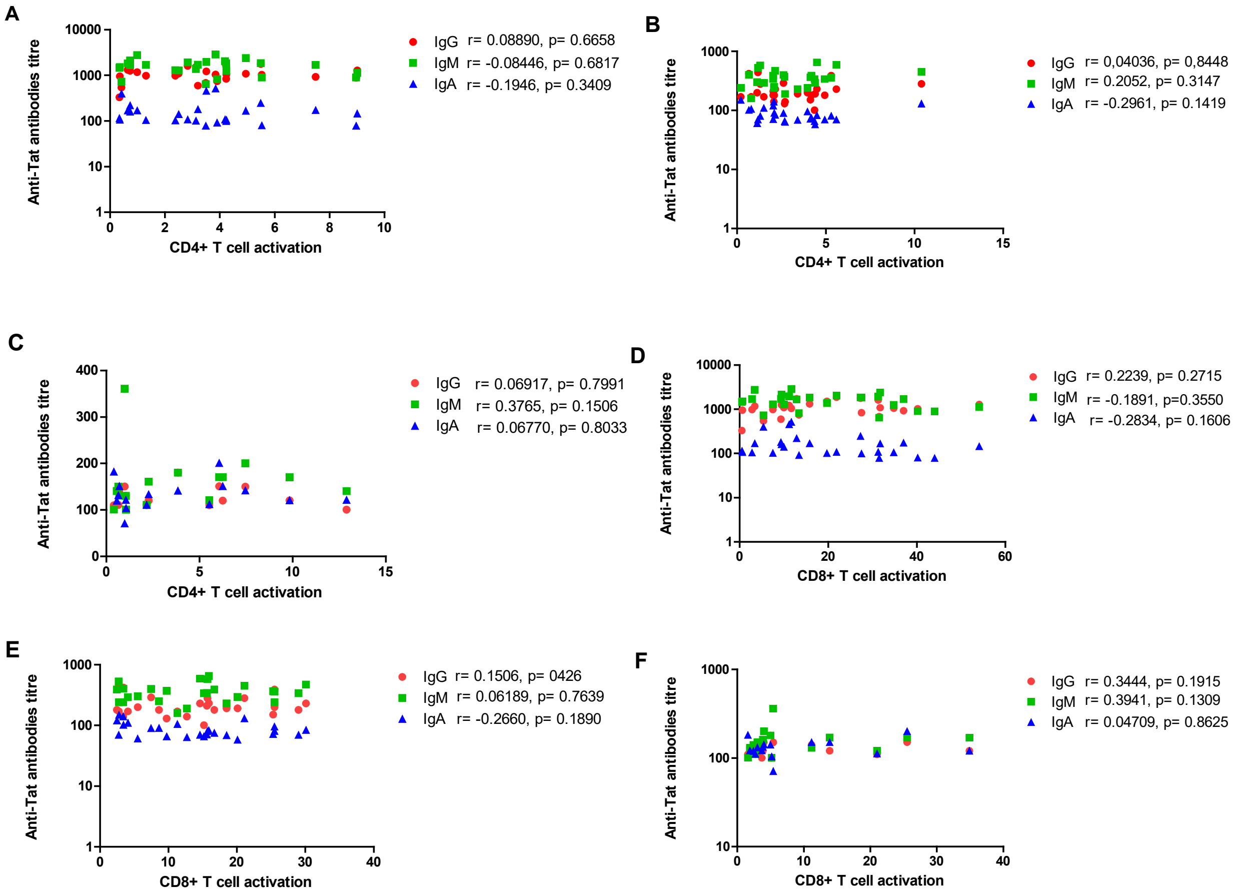

Figure 10: Association between anti-Tat antibodies isotypes and (A- C) CD4+ T and (D- F) CD8+ T cell activation. Subgroups were compared between different anti-Tat antibodies isotypes at (a and d) day 1, (b and e) 3 months and (c and f) 12 months of treatment. Y-axes represent Anti-Tat Antibodies titre and X-axes represent T cell activation. Shapes highlighted in blue, red, and green represent IgA, IgG, and IgM, respectively. Statistics comparisons were made using the Spearman (Nonparametric correlation).

Table 1: Data showing the results of HIV-1 early treated at day 1 of treatment

			Anti-Tat antibody titres			Activation		Total HIV-1 DNA
**Participants**	**CD4 T cell count (cells/ul)**	**Virl loads (copies/ml)**	**IgA**	**IgG**	**IgM**	**CD4+ T- cells**	**CD8+ T-cells**	**(copies/ml)**
Participant 1	678	11000	281,3	569,8	1207	Nd	nd	nd
Participant 2	1211	37000	181,4	597,8	1720	3,2	9,33	nd
Participant 3	634	460	170,9	1320	1834	0,65	15,8	nd
Participant 4	505	160000	167,6	1080	2381	4,95	31,7	nd
Participant 5	768	6400	174,8	926	1698	7,49	37	nd
Participant 6	653	19	170,5	1170	2772	0,99	3,41	nd
Participant 7	526	19	162	1250	2137	0,73	9,44	nd
Participant 8	341	99000	514	1044	2843	3,85	11,7	**3590**
Participant 9	873	1000	457,4	1218	1971	3,51	11,2	**425**
Participant 10	512	8200	277,6	1029	2289	Nd	nd	nd
Participant 11	847	3700	291,2	926,9	2785	Nd	nd	nd
Participant 12	818	19	221,5	1731	1681	0,73	12,9	nd
Participant 13	738	5200	248,7	1781	1859	5,49	27,3	**1013**
Participant 14	1109	19	102,7	981,3	1297	2,38	7,53	nd
Participant 15	650	21000	92,25	743,9	824,1	3,9	13,4	nd
Participant 16	226	160000	78,66	680,6	643	3,49	31,5	**442**
Participant 17	779	6900	75,06	1043	698,3	Nd	nd	**507**
Participant 18	553	14000	78,3	885,5	892,4	8,97	44	nd
Participant 19	516	19	80,24	1029	903,9	5,53	40,2	nd
Participant 20	506	110000	145,8	1281	1124	9,01	54,1	nd
Participant 21	952	19	114,9	328,9	1462	0,34	0,52	nd

Participant 22	1097	19	111,1	738,5	1880	Nd	nd	nd
Participant 23	387	42000	86,54	1139	900,6	Nd	nd	nd
Participant 24	473	12000	106,2	1051	1255	4,24	34,8	403
Participant 25	478	7000000	99,45	836,5	1832	4,23	27,5	nd
Participant 26	414	2200000	107,1	954,5	1521	0,36	0,63	nd
Participant 27	949	6200	70,11	1092	1288	2,5	9,98	nd
Participant 28	396	1300000	57,98	1610	1928	2,83	31,3	9940
Participant 29	474	760000	70,65	436,4	981,3	Nd	nd	nd
Participant 30	631	54000	80,82	544	733,8	0,42	5,37	
Participant 31	623	17000	70,2	977,5	1694	1,31	2,79	10650
Participant 32	688	53000	86,93	1520	1373	3,13	19,7	nd
Participant 33	791	1700	70,47	1866	2036	4,19	21,9	nd
Participant 34	538	460	90,99	493	732,6	Nd	nd	nd

Table 2: Data showing the results of HIV-1 early treated at 3 months of treatment

			Anti-Tat antibody titres			T-cell activation	
**Plasma samples**	**CD4+ T-cell count (cells/ul)**	**Viral loads (copies/ml)**	**IgA**	**IgG**	**IgM**	**CD4+ T-cells**	**CD8+ T-cells**
Participant 1	678	<20	120,65	180,54	390,56	2,7	12,7
Participant 2	1211	<20	150,12	170,55	240,53	5,6	14,6
Participant 3	634	<20	102,04	420,93	400,68	0,67	3,41
Participant 4	505	<20	141,09	140,31	240,89	5,31	25,5
Participant 5	768	<20	105,58	170,73	160,2	4,14	25,3
Participant 6	653	<20	65,75	100,67	340,34	nd	nd

Participant 7	526	<20	90,675	290,12	400,3	0,21	2,77
Participant 8	341	<20	130,46	280,49	450,18	1,51	4,07
Participant 9	873	<20	60,84	200,39	300,6	1,18	2,73
Participant 10	512	<20	70,92	210,96	340,97	2,04	2,47
Participant 11	847	<20	70,335	230,13	590,49	nd	nd
Participant 12	818	<20	95,63	200,39	240,3	2,62	7,45
Participant 13	738	<20	64,165	140,13	190,13	4,95	29
Participant 14	1109	<20	82,955	230,76	650,43	1,14	5,49
Participant 15	650	<20	65,93	130,28	370,22	2,07	3,39
Participant 16	226	<20	68,93	190,58	230,18	2,1	8,61
Participant 17	779	<20	80,91	390,78	360,63	4,37	15,2
Participant 18	553	<20	75,02	180,5	390,24	10,4	21,1
Participant 19	516	<20	90,855	180,36	250,43	nd	nd
Participant 20	506	<20	70,29	180,63	340,07	3,97	25,5
Participant 21	952	<20	110,07	170,51	291,79	2,68	9,74
Participant 22	1097	<20	80,415	280,13	582,01	nd	nd
Participant 23	387	<20	70,24	440,78	530,37	nd	nd
Participant 24	473	<20	72,2	150,57	363,68	1,31	15,7
Participant 25	478	<20	58,08	190,67	294,88	3,41	18,5
Participant 26	414	<20	84,62	230,36	470,79	4,5	15,9
Participant 27	949	<20	116,43	90,585	213,74	4,4	20,1
Participant 28	396	<20	152,17	180,32	422,17	2,15	30,1
Participant 29	474	<20	120,74	110,7	421,98	nd	nd
Participant 30	631	<20	90,405	150,17	361,72	nd	nd
Participant 31	623	<20	130,95	170,96	311,91	4,19	16,7

Participant 32	688	<20	90,44	260,06	310,64	0,8	11,3
Participant 33	791	<20	73,25	200,34	352,46	2,04	15,6
Participant 34	538	<20	54.44	220,82	540,63	nd	nd

nd – not done due to the lack of samples

Table 3: Data showing the results of HIV-1 early treated at 12 months of treatment

			Anti-Tat antibody titres			T-cell activation		Total HIV-1 DNA
**PLASMA SAMPLES**	**CD4+ T-cell count (cells/ul)**	**Viral load (copies/ml)**	**IgA**	**IgG**	**IgM**	**CD4+ T-cells**	**CD8+ T-cells**	**(copies/ml)**
Participant 1	678	<20	139,86	120,24	165,79	2,27	3,85	nd
Participant 2	1211	<20	146,63	130,19	201,93	nd	nd	nd
Participant 3	634	<20	87,64	120,78	123,2	nd	nd	nd
Participant 4	505	<20	203,12	150,98	171,46	6,06	25,55	nd
Participant 5	768	<20	162,315	120,11	146,31	nd	nd	nd
Participant 6	653	<20	100,85	110,03	114,84	nd	nd	nd
Participant 7	526	<20	95,9	100,31	115,43	nd	nd	nd
Participant 8	341	<20	170,03	120,29	174,64	6,25	13,9	836
Participant 9	873	<20	180,36	110,16	100,89	0,4	1,62	224
Participant 10	512	<20	110,07	100,8	122,47	nd	nd	nd
Participant 11	847	<20	170,28	120,78	201,88	nd	nd	nd
Participant 12	818	<20	230,61	110,12	129,83	5,53	21	nd
Participant 13	738	<20	100,17	100,71	100,44	1,06	5,16	0
Participant 14	1109	<20	100,33	110,66	141,85	0,56	2,43	nd
Participant 15	650	<20	80,73	140,13	140,9	nd	nd	nd
Participant 16	226	<20	140,09	150,3	180,21	7,46	4	907

Participant 17	779	<20	116,93	110,75	173,78	0,99	5,4	2108
Participant 18	553	<20	126,78	120,6	171,24	9,84	34,9	nd
Participant 19	516	<20	170,24	105,84	182,14	nd	nd	nd
Participant 20	506	<20	121,74	130,91	134,19	1,05	1,91	nd
Participant 21	952	<20	119,57	100,89	141,18	nd	nd	nd
Participant 22	1097	<20	110,61	110,12	125,38	nd	nd	nd
Participant 23	387	<20	nd	nd	nd	nd	nd	nd
Participant 24	473	<20	142,45	155.36	159.3	nd	nd	345
Participant 25	478	<20	110,16	100,35	140,58	3,85	5	nd
Participant 26	414	<20	110,39	180,54	182,14	2,17	2,73	nd
Participant 27	949	<20	135,59	110,61	144,9	nd	nd	nd
Participant 28	396	<20	76,54	90,09	93,315	nd	nd	391
Participant 29	474	<20	nd	nd	nd	nd	nd	nd
Participant 30	631	<20	140.55	180,81	140,85	0,7	11,2	nd
Participant 31	623	<20	101.33	140,4	130,73	12,9	3,7	349
Participant 32	688	<20	100	100,08	90,99	nd	Nd	nd
Participant 33	791	<20	102.0	110,84	150,3	0,65	3,03	nd
Participant 34	538	<20	nd	nd	Nd	nd	nd	nd

Table 4: Data showing the CD4 T -T-cells, viral loads and anti-Tat antibody titres of chronic treatment naïve PLWH

**Participants**	**CD4 T cell (count cells/ul)**	**Viral load (copies/ml)**	**IgA**	**IgG+IgM**
SK-1	262	47100	384.06	1112.60
SK-2	277	24800	275.92	1124.20
SK-3	213	76900	271.73	713.00
SK-4	301	43000	175.02	515.00
SK-5	495	52900	173.71	1535.60
SK-6	349	178000	274.30	1116.60
SK-7	625	50100	277.45	1124.80
SK-8	233	178000	274.75	195.40
SK-9	468	1820	329.10	187.08
SK-10	677	42900	376.86	1116.30

Table 5: Data showing the anti-Tat antibody titres and T-cell activation of HIV-1-negative participants

	Anti-Tat antibody titres			T-cell activation	
**Participants**	**IgA**	**IgG**	**IgM**	**CD4+ T-cells**	**CD8+ T-cells**
Participant -1	74,97	101,8	147,1	0,66	1,71
Participant -2	69,03	95,72	115,8	1,16	1,46
Participant -3	85,05	95,27	140,3	1,29	13,1
Participant -4	86,31	100,2	124,2	5,12	21,6
Participant -5	83,39	99,95	133,6	3,94	17,5
Participant -6	77,58	97,74	124,1	5,87	18,8
Participant -7	80,82	92,25	132,3	3,45	7,49
Participant -8	83,84	101,3	112,8	1,21	1,95
Participant -9	71,78	121,5	823,3	0,50	0.2.
Participant -10	328,6	132,6	278,2	0.20	0.7
